# Secukinumab Demonstrates Sustained Efficacy and Safety in a Taiwanese Subpopulation With Active Ankylosing Spondylitis: Four-Year Results From a Phase 3 Study, MEASURE 1

**DOI:** 10.3389/fimmu.2020.561748

**Published:** 2020-11-26

**Authors:** Jui-Cheng Tseng, James Cheng-Chung Wei, Atul Deodhar, Ruvie Martin, Brian Porter, Suzanne McCreddin, Zsolt Talloczy

**Affiliations:** ^1^Kaohsiung Veterans General Hospital, Kaohsiung, Taiwan; ^2^Institute of Medicine, Chung Shan Medical University, Taichung, Taiwan; ^3^Department of Medicine, Chung Shan Medical University Hospital, Taichung, Taiwan; ^4^Graduate Institute of Integrated Medicine, China Medical University, Taichung, Taiwan; ^5^Oregon Health and Science University, Portland, OR, United States; ^6^Novartis Pharmaceuticals Corporation, East Hanover, NJ, United States; ^7^Clinical Development, Novartis Ireland Limited, Dublin, Ireland

**Keywords:** biologics, interleukin-17A inhibitor, Taiwan, secukinumab, ankylosing spondylitis

## Abstract

**Objectives:**

To present the long-term (4-year) efficacy and safety of secukinumab in Taiwanese patients with active AS in the MEASURE 1 extension study.

**Methods:**

This *post hoc* analysis reports data from Taiwanese patients originally randomized to subcutaneous secukinumab 150 or 75mg or placebo every 4 weeks (following intravenous loading dose) who were invited to enter the 3-year extension study. Assessments at Week 208 included ASAS20/40 responses and other clinically relevant endpoints. Efficacy data are presented as observed. Safety analyses included all patients who received ≥1 dose of secukinumab.

**Results:**

Of the 57 Taiwanese patients in the core trial, 48 entered the extension study and 87.5% patients (42/48) completed 4 years of treatment. Thirteen Taiwanese patients (including placebo-switchers) were escalated from 75 to 150mg (approved dose) at some point starting from Week 172. ASAS20/40 responses were sustained through 4 years in the Taiwanese patients who were originally randomized to secukinumab 150mg. Clinical responses were improved in those patients who received dose-escalation from 75 to 150mg during the study. No unexpected safety signals were reported.

**Conclusion:**

Secukinumab 150mg demonstrated sustained efficacy over 4 years in Taiwanese patients with active ankylosing spondylitis. The safety profile of secukinumab was consistent with previous reports.

**ClinicalTrials.gov identifier:**

NCT01863732.

## Introduction

Ankylosing spondylitis (AS) is a chronic inflammatory arthritis that belongs to the family of spondyloarthritides and is clinically characterized by sacroiliitis, spinal ankylosis, extra-articular manifestations, and enthesitis (1, 2). AS causes progressive and irreversible structural damage of the sacroiliac and spinal joints, disability, and reduced quality of life (QoL) (3).

The prevalence of AS in the Chinese population is reported to be 0.3 to 0.5% (4, 5). A community-based epidemiologic study in Taiwan observed that the prevalence of AS in Taiwan Chinese was 0.38% (6).

The Assessment of SpondyloArthritis International Society (ASAS), the European League Against Rheumatism (EULAR), the American College of Rheumatology (ACR)/Spondylitis Association of America, and the Spondyloarthritis Research and Treatment Network (SPARTAN) treatment guidelines recommend non-steroidal anti-inflammatory drugs (NSAIDs) as the first-line treatment for active AS, followed by anti-tumor necrosis factor (TNF) agents or interleukin (IL)-17 inhibitors for patients who are nonresponsive to NSAIDs (2, 7). Currently, anti-TNF agents and IL-17 inhibitors are the only classes of biologics approved for the treatment of AS in Asia and worldwide (8). Up to 40% of AS patients experience an inadequate response or intolerance (IR) to anti-TNF therapies, and cases of relapse have also been reported, specifically in Asian patients, after completion of short-term treatment with anti-TNF therapies (9–12). Long-term studies with anti-TNF agents have shown a high incidence of tuberculosis and hepatitis B virus infections, as well as immunogenicity in Asian patients with AS (13–15).

Secukinumab, a fully human monoclonal immunoglobulin G (IgG)1κ antibody that directly inhibits IL-17A (expressed in a recombinant Chinese Hamster Ovary (CHO) cell line), has demonstrated sustained efficacy with a favorable and consistent safety profile through 5-years of treatment (16–19). Here we present the long-term (4-year) efficacy and safety data for secukinumab in a cohort of Taiwanese patients with active AS from the MEASURE 1 extension study.

## Materials and Methods

### Study Design and Patients

MEASURE 1 is a Phase 3, randomized, placebo-controlled 2-year study (NCT01358175) (16) with a 3-year extension trial (NCT1863732) (19). The study was double-blinded until Week 156 (3 years) followed by open-label treatment up to Week 260 (5 years). The Taiwanese subpopulation was assessed through Week 208 (4 years) as an interim analysis. The 52-week (1-year) results for the pooled Asian subpopulation from MEASURE 1 (NCT01358175) and MEASURE 2 (NCT01649375) phase 3 studies having 63 and 9 patients, respectively, have been reported previously (12).

In MEASURE 1, patients were initially randomized to receive intravenous (i.v.) secukinumab 10 mg/kg at baseline and Weeks 2 and 4, followed by either subcutaneous (s.c.) secukinumab 150 mg (i.v → 150 mg) or 75 mg (i.v. → 75 mg) every 4 weeks thereafter. Matched placebo was given on the same i.v. to s.c. dosing schedule, and placebo patients were re-randomized to secukinumab 150 mg s.c. or secukinumab 75 mg s.c. (hereafter referred to as placebo-switchers) by Week 16 (ASAS20 non-responders at Week 16) or Week 24 (ASAS20 responders at Week 16). After the 2-year core trial, patients receiving secukinumab 150 mg or 75 mg s.c. were invited to enter the 3-year extension trial. After protocol amendment, the study medication for patients on the secukinumab 75 mg treatment arm could be escalated to 150 mg every 4 weeks for patients whose overall therapeutic response was not fully achieved and could improve with a higher dose, as judged by the investigator. The dose escalation of the study medication could be determined at any site visit. For patients escalated to secukinumab 150 mg s.c. every 4 weeks, no dose reduction was performed and if the patient was unable to tolerate the 150 mg dose of secukinumab, alternative treatment options were considered only after discontinuation from the study. The first Taiwanese patient was dose escalated from secukinumab 75 mg to 150 mg at Week 172 whereas the first patient to receive escalated dose in the overall population was at Week 168 (as reported previously) (18). Patients were stratified according to previous anti-TNF therapy [patients who were naïve to anti-TNF therapy (anti-TNF-naïve) or those with a history of inadequate response or intolerance to no more than one of these agents (anti-TNF-IR)].

Detailed patient eligibility criteria have been previously reported (16). Briefly, patients aged ≥ 18 years with AS fulfilling the modified New York criteria with a score ≥ 4 (0–10) on the Bath Ankylosing Spondylitis Disease Activity Index (BASDAI) and a spinal pain score ≥ 40 mm on a 0–100 mm visual analog scale, despite treatment with maximum tolerated doses of NSAIDs, were included in the core study. Concomitant sulfasalazine (≤ 3 g/day), methotrexate (≤ 25 mg/week), prednisone or equivalent (≤ 10 mg/day) and NSAIDs were permitted at stable doses. Patients were excluded if they had total spinal ankylosis, evidence of infection or malignancy on chest X-ray, or a history of human immunodeficiency virus or hepatitis B/C infection at screening. Patients with active systemic infection within 2 weeks prior to randomization or previous treatment with cell-depleting therapies or biologics other than anti-TNF therapy were also excluded. This study was conducted in accordance with the Declaration of Helsinki and was approved by the institutional review board or independent ethics committee at each participating center. Written informed consent was obtained from all enrolled patients.

### Outcome and Assessments

Assessments through Week 208 (4 years) included ASAS20 response (defined as an improvement of ≥ 20% and absolute improvement of ≥ 1 unit on a scale of 10 in at least three of the four main ASAS domains and no worsening of ≥ 20% and ≥ 1 unit on a scale of 10 in the remaining domain), ASAS40 response (defined as an improvement of ≥ 40% and absolute improvement of ≥ 2 units on a scale of 10 in at least three of the four main ASAS domains and no worsening at all in the remaining domain), the proportion of patients achieving Ankylosing Spondylitis Disease Activity Score-C-reactive protein Inactive Disease (ASDAS-CRP ID), and mean change from baseline in high sensitivity CRP (hsCRP), BASDAI, Maastricht Ankylosing Spondylitis Enthesitis Score (MASES), Bath Ankylosing Spondylitis Functional Index (BASFI), Bath Ankylosing Spondylitis Metrology Index (BASMI), Functional Assessment of Chronic illnesses Therapy-Fatigue (FACIT-Fatigue), Ankylosing Spondylitis Quality of Life (ASQoL), and Short Form Survey-36 physical component summary (SF-36 PCS). ASAS5/6, BASDAI50 (50% improvement from baseline in BASDAI), and ASAS partial remission responses were also assessed.

### Statistical Analysis

The sample size calculation and analysis of the primary and other efficacy endpoints have been reported previously for the overall study population (16). This *post hoc* analysis reports data from a Taiwanese patient subpopulation (N=57) who were initially randomized to the core trial and who continued in the extension trial (N=48) to Week 208 (4 years). Clinical outcomes are reported for Taiwanese patients originally randomized to secukinumab 150 or 75 mg, but not to placebo, to show the full 4-year efficacy of treatment, as well as separately for all Taiwanese patients who entered the extension study, i.e., including patients originally randomized to secukinumab and placebo switchers (hereafter, referred as the Any secukinumab 150 mg and Any secukinumab 75 mg groups). Efficacy data are presented as observed.

Safety analyses were pooled for both doses and included all Taiwanese patients who received ≥1 dose of secukinumab at any time throughout the core or extension trials. Descriptive statistics for observed safety data are provided.

## Results

### Patients

**Figure 1:**
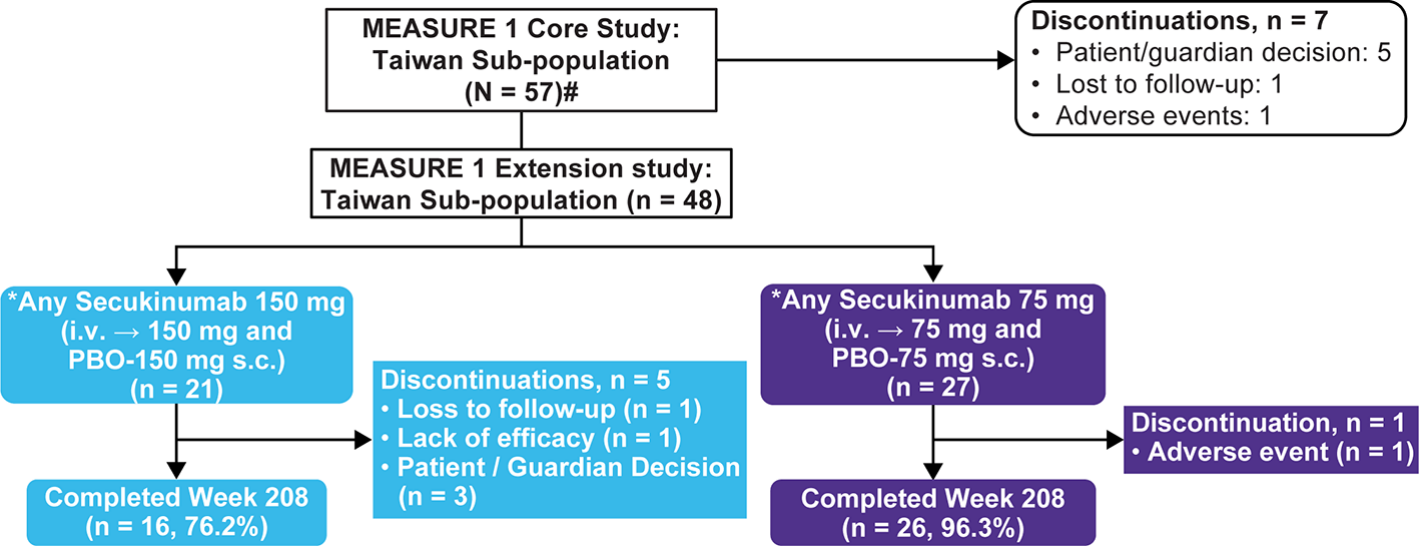
Patient disposition through 4 years. N, number of patients randomized; n, number of patients in a specific category i.v., Intravenous; s.c., Subcutaneous; PBO, Placebo. ^#^Includes two Taiwanese patients who did not enter the extension phase. ^*^Includes placebo-switchers.

Of the 371 total randomized patients in the core study, 57 (~16%) were of Taiwanese origin. Overall 84% (48/57) Taiwanese patients completed the 2-year core trial and chose to enter the extension study with 21 (43.8%) and 27 (56.3%) patients in the Any secukinumab 150 mg and 75 mg groups, respectively. The overall retention rate at Week 208 was 87.5% (42/48) **(****Figure 1****)**. A total of 5 patients discontinued in the Any secukinumab 150 mg group (3 due to patient decision, 1 due to lack of efficacy, and 1 was lost to follow-up); 1 patient discontinued in the Any secukinumab 75 mg group due to an adverse event (AE). A total of 13 Taiwanese patients (including placebo-switchers) dose-escalated from secukinumab 75 mg to 150 mg (approved dose) at various time points starting from Week 172.

Baseline demographic and disease characteristics were generally similar across the secukinumab and placebo groups in the Taiwanese subpopulation to that of the overall population, except for lower hsCRP levels and a higher percentage of HLA-B27 positive patients in the Taiwanese subpopulation (**Table 1** and **Supplementary Table S1**). A total of 12.5% patients were inadequate responders to previous anti-TNF treatment.

**Table 1:** Baseline demographic and clinical characteristics.

Characteristic	Secukinumab 150 mg(N = 13)	Secukinumab75 mg(N = 19)	Placebo-secukinumab150 mg(N = 8)	Placebo-secukinumab75 mg(N = 8)
**Age (years), mean (SD)**	32.6 (9.55)	34.1 (11.17)	43.4 (11.10)	33.6 (8.53)
**Male, n (%)**	10 (76.9)	16 (84.2)	5 (62.5)	8 (100.0)
**Weight (kg), mean (SD)**	67.35 (11.77)	66.34 (9.69)	77.50 (17.74)	70.50 (13.69)
**Total back pain (0–100 mm), mean (SD)**	65.46 (14.89)	58.42 (13.13)	66.0 (13.26)	56.13 (12.61)
**BASDAI (total), mean (SD)**	6.67 (1.17)	5.77 (1.08)	6.87 (1.44)	5.95 (1.39)
**hsCRP (mg/L), median (Min – Max)**	5.20 (2.2–52.6)	8.10 (0.6–51.8)	3.80 (0.6–25.9)	3.80 (0.2–15.9)
**HLA-B27 positive, n (%)**	13 (100.0)	18 (94.7)	8 (100.0)	8 (100.0)
**Mean time since AS diagnosis (years), mean (SD)**	8.38 (4.49)	10.01 (5.13)	9.81 (5.95)	12.55 (8.61)
**Anti–TNF-naïve, n (%)**	12 (92.3)	15 (78.9)	8 (100.0)	7 (87.5)

BASDAI, Bath Ankylosing Spondylitis Disease Activity Index; hsCRP, High Sensitivity C-reactive Protein; HLA, Human Leukocyte Antigen; SD, Standard Deviation; TNF, Tumor Necrosis Factor.

### Efficacy

Efficacy results from the core study for the overall population have been published previously (primary endpoint of ASAS20 response at Week 16: 61% with secukinumab 150 mg vs. 29% with placebo, P < 0.001) (16).

### Efficacy in Originally Randomized Patients to Secukinumab (Excluding Placebo-Switchers)

**Figure 2:**
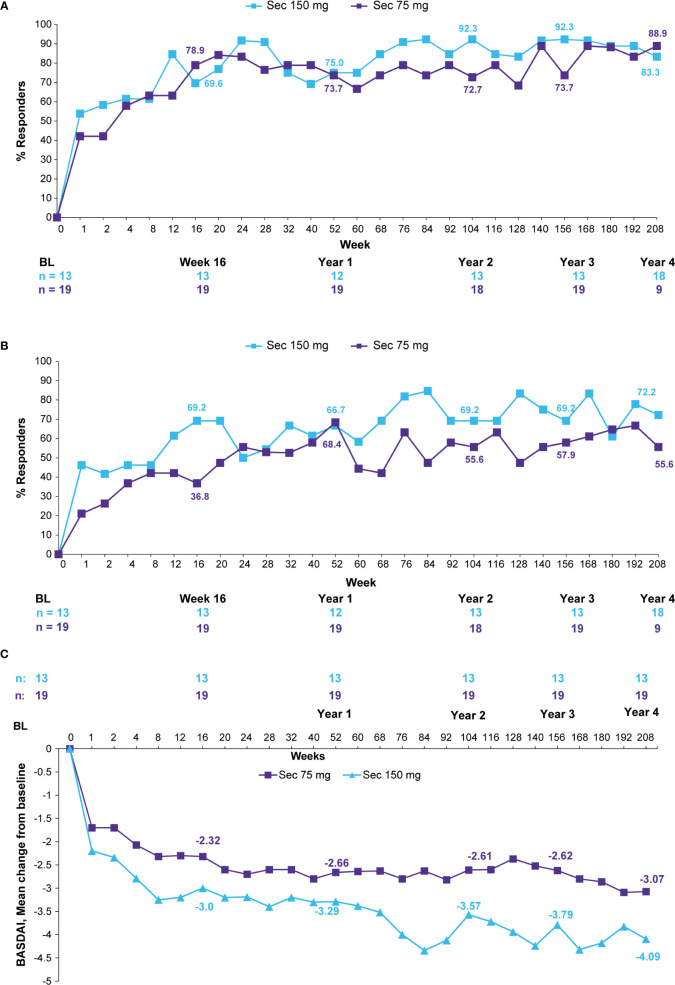
**(A)** ASAS20 responses, **(B)** ASAS40 responses, and **(C)** BASDAI mean change from baseline through 4 years (observed data for patients originally randomized to secukinumab). Observed data presented through Week 208. n, number of evaluable patients. The secukinumab 75 mg group included 9 patients who were dose-escalated from 75 to 150 mg at any time point starting from Week 172. Number of patients shown for Baseline, Weeks 16, 52 (Year 1), 104 (Year 2), 156 (Year 3), and 208 (Year 4). ASAS, Assessment of Spondyloarthritis International Society; BASDAI, Bath Ankylosing Spondylitis Disease Activity Index; Sec, Secukinumab.

ASAS20/40 responses were sustained through 4 years in the Taiwanese patients who were originally randomized to secukinumab 150 mg or 75 mg **(****Figure 2A****)**. ASAS20/40 responses were 83.3% each at 4 years in the secukinumab 150 mg group. In the secukinumab 75 mg group which included 9 patients who had dose-escalated to 150 mg, ASAS20/40 responses at 4 years for secukinumab 150 mg were 88.9% and 72.2%, respectively **(****Figure 2B****)**. BASDAI scores and ASDAS-CRP ID clinical responses were sustained or further improved in the secukinumab 150 mg group through 4 years (**Figure 2C** and **Table 2**).

**Table 2:** Clinical improvements with secukinumab through Week 208 (observed data for patients originally randomized to secukinumab).

Variable	Week	Secukinumab 150 mg (N = 13)	Secukinumab 75 mg (N = 19)
**ASDAS-CRP inactive disease, % (n)**	**16**	15.4 (13)	10.5 (19)
	**52**	16.7 (12)	21.1 (19)
	**104**	30.8 (13)	16.7 (18)
	**156**	30.8 (13)	26.3 (19)
	**208**	41.7 (12)	27.8 (18)
**hsCRP, mean change from baseline ± SD (n)**	**16**	−10.5 ± 16.29 (13)	−8.2 ± 15.38 (19)
	**52**	−9.1 ± 15.97 (12)	−8.2 ± 12.09 (19)
	**104**	−9.6 ± 13.69 (13)	−7.5 ± 11.83 (18)
	**156**	−9.4 ± 13.22 (13)	−8.3 ± 12.35 (19)
	**208**	−10.2 ± 16.92 (12)	−10.2 ± 15.76 (18)
**SF-36 PCS, mean change from baseline ± SD (n)**	**16**	5.7 ± 5.31 (13)	6.7 ± 4.42 (19)
	**52**	7.3 ± 6.99 (13)	7.84 ± 4.76 (19)
	**104**	7.4 ± 6.97 (13)	7.7 ± 5.15 (18)
	**156**	6.9 ± 7.39 (13)	7.1 ± 5.58 (19)
	**208**	7.9 ± 6.57 (12)	9.4 ± 4.76 (18)
**ASQoL, mean change from baseline ± SD (n)**	**16**	−2.5 ± 3.45 (13)	−4.1 ± 3.30 (19)
	**52**	−3.5 ± 4.70 (13)	−4.1 ± 3.74 (19)
	**104**	−3.6 ± 4.96 (13)	−4.7 ± 4.21 (18)
	**156**	NA	NA
	**208**	NA	NA
**MASES, mean change from baseline ± SD (n)**	**16**	−0.6 ± 0.96 (13)	−0.7 ± 2.16 (19)
	**52**	−0.8 ± 1.01 (13)	−0.9 ± 2.15 (19)
	**104**	−0.8 ± 1.01 (13)	−1.1 ± 2.56 (18)
	**156**	−0.6 ± 1.26 (13)	−1.3 ± 1.97 (19)
	**208**	−0.9 ± 1.16 (12)	−1.5 ± 2.33 (18)
**BASFI, mean change from baseline ± SD (n)**	**16**	−2.3 ± 2.06 (13)	−2.0 ± 1.32 (19)
	**52**	−2.0 ± 2.18 (12)	−2.3 ± 1.70 (19)
	**104**	−2.9 ± 2.07 (13)	−2.2 ± 1.87 (18)
	**156**	−2.8 ± 2.0 (13)	−2.4 ± 1.86 (19)
	**208**	−2.8 ± 2.13 (12)	−2.4 ± 1.59 (18)
**BASMI, mean change from baseline ± SD (n)**	**16**	−0.06 ± 0.60 (13)	−0.23 ± 0.87 (19)
	**52**	−0.2 ± 0.54 (12)	−0.3 ± 0.83 (19)
	**104**	−0.02 ± 0.62 (13)	−0.26 ± 0.81 (18)
	**156**	−0.14 ± 0.65 (13)	−0.12 ± 0.80 (19)
	**208**	−0.17 ± 0.56 (12)	−0.1 ± 0.95 (18)
**FACIT-Fatigue, mean change from baseline ± SD (n)**	**16**	8.2 ± 8.83 (13)	6.3 ± 6.50 (19)
	**52**	9.3 ± 11.83 (13)	8.2 ± 5.37 (19)
	**104**	11.4 ± 9.89 (13)	8.8 ± 7.55 (18)
	**156**	10.9 ± 10.80 (13)	8.6 ± 8.83 (19)
	**208**	11.5 ± 12.10 (12)	9.9 ± 7.45 (18)
**ASAS5/6 response, % (n)**	**16**	61.5 (13)	68.4 (19)
	**52**	58.3 (12)	52.6 (19)
	**104**	69.2 (13)	55.6 (18)
	**156**	69.3 (13)	63.2 (19)
	**208**	66.7 (12)	83.3 (18)
**BASDAI50 response, % (n)**	**16**	53.8 (13)	36.8 (19)
	**52**	58.3 (12)	47.4 (19)
	**104**	61.5 (13)	38.9 (18)
	**156**	53.8 (13)	47.4 (19)
	**208**	75 (12)	55.6 (18)
**ASAS partial remission response, % (n)**	**16**	7.7 (13)	10.5 (19)
	**52**	16.7 (12)	15.8 (19)
	**104**	23.1 (13)	22.2 (18)
	**156**	15.4 (13)	10.5 (19)
	**208**	25 (12)	16.7 (18)

Data shown are as observed through 4 years;

N, number of patients randomized, n, number of patients assessed.

NA, not assessed (data not collected)/not applicable.

At Week 208, the secukinumab 75 mg group included patients (n= 9) who were dose-escalated from 75 mg to 150 mg starting from Week 172.

hsCRP, high sensitivity C-reactive Protein; ASDAS, Ankylosing Spondylitis Disease Activity Score; SF-36 PCS, Short Form Survey-36 Physical Component Summary; ASQoL, Ankylosing Spondylitis Quality of Life; MASES, Maastricht Ankylosing Spondylitis Enthesitis Score; BASFI, Bath Ankylosing Spondylitis Functional Index; BASMI, Bath Ankylosing Spondylitis Metrology Index; FACIT, Functional Assessment Of Chronic Illness Therapy; BASDAI, Bath Ankylosing Spondylitis Disease Activity Index, ASAS, Assessment of Spondyloarthritis International Society.

**Figure 3:**
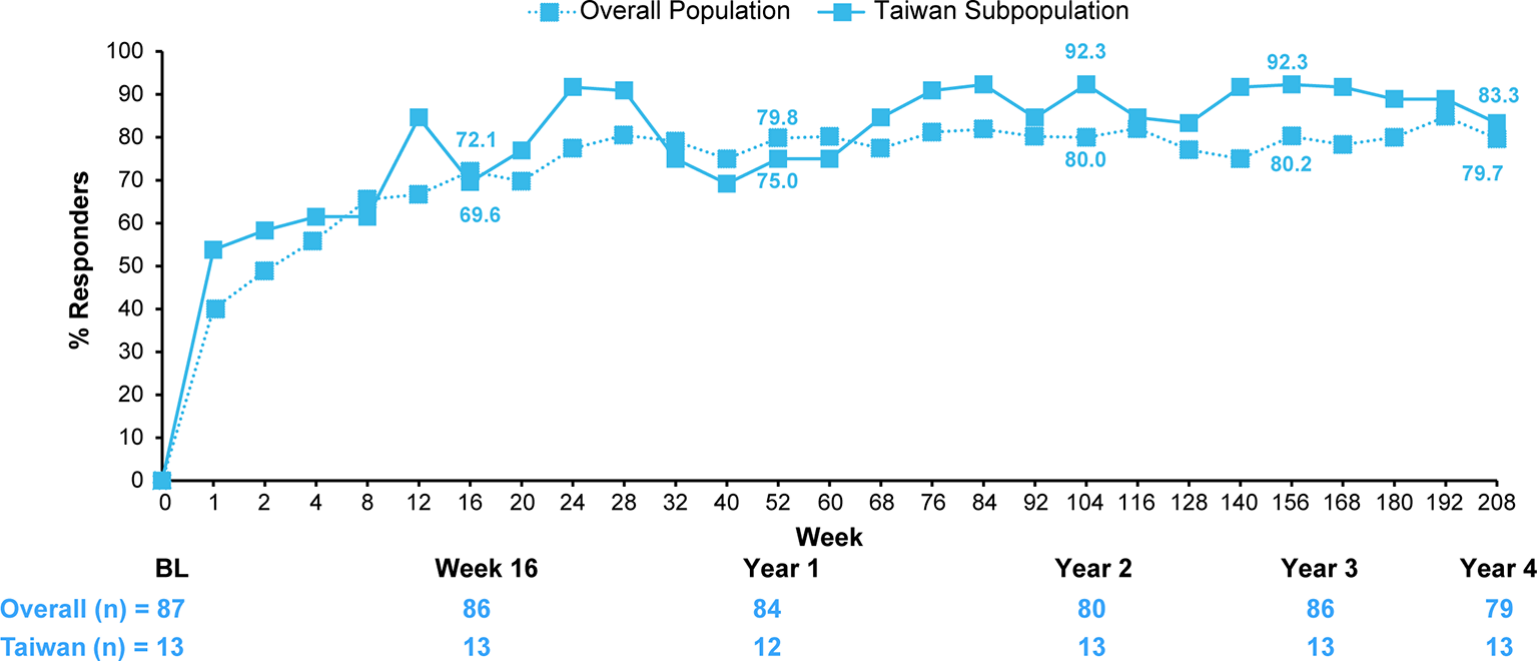
ASAS20 response of patients originally randomized to secukinumab 150 mg in the overall population versus the Taiwanese subpopulation. Data shown are as observed through 4 years; n, number of evaluable patients. Number of patients shown for Baseline, Weeks 16, 52 (Year 1), 104 (Year 2), 156 (Year 3), and 208 (Year 4). For overall population, the secukinumab 75 mg group included 25 patients who were dose-escalated from 75 mg to 150 mg starting from Week 168. For the Taiwanese subpopulation, the secukinumab 75 mg group included 9 patients who were dose-escalated from 75 mg to 150 mg starting from Week 172.

A comparison of efficacy outcomes in the overall population of patients originally randomized secukinumab 150 mg versus those of the Taiwanese subpopulation through 4 years are presented in **Figure 3** and **Supplementary Table S2**.

### Efficacy in All Patients (Including Placebo-Switchers)

In the overall group of Taiwanese patients who received secukinumab 150 mg and 75 mg (i.e., including patients who switched from placebo to secukinumab at Week 16/24) efficacy was sustained through 4 years with similar responses to those observed in the originally randomized groups **(****Supplementary Table S3**).

### Efficacy Following Dose Escalation

A total of 13 out of the 27 (48.1%) patients who entered the extension study on 75 mg dose-escalated to 150 mg at some point starting from Week 172. Clinical responses were improved across most efficacy endpoints in patients who dose-escalated **(****Supplementary Table S3**).

### Safety

Rates of the most frequent treatment-emergent AEs (TEAEs) and selected AEs of interest in the Taiwanese subpopulation are shown in **Table 3**. Over the entire treatment period and combined for both secukinumab 150 mg and 75 mg doses, the exposure-adjusted incidence rates (EAIR) of TEAEs and serious adverse events (SAEs) were 251.9 and 3.4 events per 100 patient-years, respectively. Most of these AEs were mild to moderate. The most commonly reported TEAEs were dyslipidaemia (EAIR = 12.3) and viral upper respiratory tract infection (EAIR = 11.0). There were no cases of reactivation of hepatitis B or tuberculosis reported. Major adverse cardiovascular events including myocardial infarction (MI), stroke or cardiovascular death were reported in 1 patient (EAIR = 0.5). No cases of inflammatory bowel disease, including Crohn’s disease or ulcerative colitis, were reported. Uveitis was reported in 5 subjects (8.9%) (2 cases were *de novo* while 3 had flares out of the 14 patients who had pre-existing medical history of uveitis). The immunological assays were conducted for MEASURE 1 study and none of the Taiwanese patients had any treatment emergent anti-drug antibodies during the study. No cases of treatment-emergent suicidality-related AEs were reported during the entire treatment period.

**Table 3:** Consolidated clinical safety for secukinumab doses during the entire treatment period.

Variable	Any secukinumab 150 mg and 75 mg^*^N = 56
Any AE, n (EAIR per 100 pt. years)	51 (251.9)
Any SAE, n (EAIR per 100 pt. years)	6 (3.4)
Discontinuation due to AE, n (%)	2 (3.6)
**Most Frequent AEs, n (%)**	
Dyslipidaemia	17 (12.3)
Viral upper respiratory tract infection	17 (11.0)
Leukopenia	13 (23.2)
Mouth ulceration	13 (23.2)
Upper respiratory tract infection	13 (23.2)
Pharyngitis	11 (9.6)
**Selected AEs of interest, n (EAIR per 100 patient-years)**	
Serious infections	2 (1.0)
Uveitis	5 (2.7)
Malignant/unspecified tumors	3 (1.6)
Major adverse cardiac events	1 (0.5)

AE, adverse event; EAIR, exposure-adjusted incidence rate; SAE, serious adverse event.

^*^Includes one Taiwanese subject who did not enter extension study but had at least one dose of secukinumab during the core study.

Events listed according to preferred term in the Medical Dictionary for Regulatory Activities (MedDRA) version 20.0, sorted in descending order of EAIR in the Any secukinumab 150 and 75 mg group for the entire treatment period.

## Discussion

While the efficacy and safety of secukinumab in AS is well documented, (12, 16, 19–21) this is the first study demonstrating the long-term (4-year) efficacy, safety, and tolerability of secukinumab in Taiwanese patients with active AS.

These long-term results demonstrated that secukinumab 150 mg conferred sustained efficacy with a favorable and consistent safety profile over 4 years of treatment in Taiwanese patients with active AS. These findings are in line with previously reported 52-week pooled results in an Asian subpopulation from the MEASURE 1 and MEASURE 2 studies (12) and are consistent with previous long-term reports of the overall MEASURE 1 study population results (16–18, 22).

Overall, the study reported high retention rates. Clinical improvements were observed in ASAS20 response and in higher hurdle clinical endpoints, such as ASAS40, BASDAI50, and ASDAS-CRP ID with the approved secukinumab 150 mg dose that showed consistently better efficacy at most time points. These results were generally consistent with the overall population. Mean change from baseline in BASDAI score also showed higher improvement with secukinumab 150 mg than 75 mg at most time points. Improvements were also sustained in physical function (BASFI, SF-36 PCS), spinal mobility (BASMI), fatigue (FACIT-Fatigue) and QoL (ASQoL) over 4 years, further supporting the long-term efficacy of secukinumab in Taiwanese patients with AS. Following a protocol amendment, 48% of Taiwanese patients (13/27) receiving secukinumab 75 mg up-titrated to 150 mg during the study based on the clinical judgement of the treating physician, highlighting that 150 mg was a more efficacious dose for approximately half of the Taiwanese subpopulation.

The rates of SAEs and AEs leading to discontinuation were low with secukinumab. Secukinumab was well tolerated over 4 years with a safety profile consistent with previous reports in the overall MEASURE 1 population as well as pooled safety analysis of 21 secukinumab clinical trials across different indications (22, 23). Thus, the efficacy and safety of secukinumab in Taiwanese patients were observed to be similar to the non-Taiwanese population. The limitation of this *post hoc* analysis was the limited sample size and subsequent lack of statistical power to demonstrate the superiority of secukinumab versus placebo in Taiwanese patients. In addition, the MEASURE 1 study was not designed or powered to evaluate Taiwanese patients alone. Therefore, these results must be interpreted with caution.

In conclusion, secukinumab provided sustained long-term efficacy for the treatment of active AS in Taiwanese patients through 4 years. Secukinumab was well tolerated with a consistent safety profile through 4 years, with no new or unexpected safety risks identified. Overall, these long-term results support secukinumab 150 mg s.c. in the treatment of Taiwanese patients with active AS.

## Data Availability Statement

The datasets generated and/or analyzed during the current study are not publicly available. Novartis is committed to sharing with qualified external researchers access to patient-level data and supporting clinical documents from eligible studies. These requests are reviewed and approved based on scientific merit. All data provided are anonymized to respect the privacy of patients who have participated in the trial in line with applicable laws and regulations. The data may be requested from the corresponding author of the manuscript.

## Ethics Statement

The studies involving human participants were reviewed and approved by institutional review board or independent ethics committee at each participating center. The patients/participants provided their written informed consent to participate in this study.

## Author Contributions

All named authors meet the International Committee of Medical Journal Editors (ICMJE) criteria for authorship of this article, take responsibility for the integrity of the work as a whole, and were involved in the drafting and critical review of the manuscript. All authors agree to be accountable for all aspects of the work and attest to the accuracy and integrity of the work. All authors contributed to the article and approved the submitted version.

## Funding

The clinical study was sponsored by Novartis Pharma AG, Basel, Switzerland, and designed by the scientific steering committee and Novartis personnel. Novartis funded the medical writing support.

## Conflict of Interest

J-CT received honoraria for consulting or speaking for, or has received research grants from Pfizer, AbbVie, Jansen, UCB, Chugai, Roche, Bristol Myer Squibb (BMS), Eli Lilly, Novartis and Boehringer Ingelheim. JW received honoraria for consultation from TSH biopharm, Abbvie, BMS, Celgene, Chugai, Eisai, Janssen, Novartis, Pfizer, Sanofi-Aventis, and UCB pharma and research grant support from Abbvie, BMS, Celgene, Eli Lilly, Janssen, Novartis, Pfizer, and UCB. AD received honoraria for consulting or speaking for, or has received research grants from AbbVie, Amgen, Boehringer Ingelheim, Bristol Myer Squibb (BMS), Eli Lilly, Glaxo Smith & Kline (GSK), Janssen, Novartis, Pfizer, and UCB. RM is an employee of Novartis with Novartis stock. BP is an employee of Novartis with Novartis stock. SM is an employee of Novartis with Novartis stock. ZT is an Employee of Novartis with Novartis stock.

## References

[1] Braun J Sieper J Ankylosing spondylitis. Lancet (2007) 369(9570):1379–90. 10.1016/S0140-6736(07)60635-7 17448825

[2] Ward MM Deodhar A Akl EA Lui A Ermann J Gensler LS American College of Rheumatology/Spondylitis Association of America/Spondyloarthritis Research and Treatment Network 2015 Recommendations for the Treatment of Ankylosing Spondylitis and Nonradiographic Axial Spondyloarthritis. Arthritis Rheumatol (Hoboken NJ) (2016) 68(2):282–98. 10.1002/art.39298 PMC5123840 26401991

[3] Schett G Coates LC Ash ZR Finzel S Conaghan PG Structural damage in rheumatoid arthritis, psoriatic arthritis, and ankylosing spondylitis: traditional views, novel insights gained from TNF blockade, and concepts for the future. Arthritis Res Ther (2011) 13 Suppl 1(Suppl 1):S4–S. 10.1186/1478-6354-13-S1-S4 PMC3123965 21624183

[4] Chou CT Chen JM Hsu CM Chen SJ HLA-B27 and its subtypes in 4 Taiwanese Aborigine tribes: a comparison to Han Chinese patients with ankylosing spondylitis. J Rheumatol (2003) 30(2):321–5. 12563689

[5] Feltkamp TE Mardjuadi A Huang F Chou CT Spondyloarthropathies in eastern Asia. Curr Opin Rheumatol (2001) 13(4):285–90. 10.1097/00002281-200107000-00007 11555729

[6] Ho HH Chen JY Ankylosing spondylitis: Chinese perspective, clinical phenotypes, and associated extra-articular systemic features. Curr Rheumatol Rep (2013) 15(8):344. 10.1007/s11926-013-0344-0 23797415

[7] van der Heijde D Ramiro S Landewe R Baraliakos X Van den Bosch F Sepriano A update of the ASAS-EULAR management recommendations for axial spondyloarthritis. Ann Rheum Dis (2017) 76(6):978–91. 10.1136/annrheumdis-2016-210770 28087505

[8] Cheung PP Anti-IL17A in Axial Spondyloarthritis-Where Are We At? Front Med (Lausanne) (2017) 4:1. 10.3389/fmed.2017.00001 28149838 PMC5241317

[9] Accortt NA Bonafede MM Collier DH Iles J Curtis JR Risk of Subsequent Infection Among Patients Receiving Tumor Necrosis Factor Inhibitors and Other Disease-Modifying Antirheumatic Drugs. Arthritis Rheumatol (Hoboken NJ) (2016) 68(1):67–76. 10.1002/art.39416 26359948

[10] Sampaio-Barros PD van der Horst-Bruinsma IE Adverse effects of TNF inhibitors in SpA: are they different from RA? Best Pract Res Clin Rheumatol (2014) 28(5):747–63. 10.1016/j.berh.2014.10.001 25488782

[11] Zhang S Li Y Deng X Huang F Similarities and differences between spondyloarthritis in Asia and other parts of the world. Curr Opin Rheumatol (2011) 23(4):334–8. 10.1097/BOR.0b013e32834640a9 21494185

[12] Wei JC Baeten D Sieper J Deodhar A Bhosekar V Martin R Efficacy and safety of secukinumab in Asian patients with active ankylosing spondylitis: 52-week pooled results from two phase 3 studies. Int J Rheum Dis (2017) 20(5):589–96. 10.1111/1756-185x.13094 28544533

[13] Jung SM Kim HS Kim HR Kim NY Lee JH Kim J Immunogenicity of anti-tumour necrosis factor therapy in Korean patients with rheumatoid arthritis and ankylosing spondylitis. Int Immunopharmacol (2014) 21(1):20–5. 10.1016/j.intimp.2014.04.006 24752013

[14] Montiel PM Solis JA Chirinos JA Casis B Sanchez F Rodriguez S Hepatitis B virus reactivation during therapy with etanercept in an HBsAg-negative and anti-HBs-positive patient. Liver International: Off J Int Assoc Study Liver (2008) 28(5):718–20. 10.1111/j.1478-3231.2007.01665.x 18433400

[15] Navarra SV Tang B Lu L Lin HY Mok CC Asavatanabodee P Risk of tuberculosis with anti-tumor necrosis factor-alpha therapy: substantially higher number of patients at risk in Asia. Int J Rheum Dis (2014) 17(3):291–8. 10.1111/1756-185x.12188 PMC4034594 24131578

[16] Baeten D Sieper J Braun J Baraliakos X Dougados M Emery P Secukinumab, an Interleukin-17A Inhibitor, in Ankylosing Spondylitis. N Engl J Med (2015) 373(26):2534–48. 10.1056/NEJMoa1505066 26699169

[17] Braun J Baraliakos X Deodhar A Baeten D Sieper J Emery P Effect of secukinumab on clinical and radiographic outcomes in ankylosing spondylitis: 2-year results from the randomised phase III MEASURE 1 study. Ann Rheum Dis (2017) 76(6):1070–7. 10.1136/annrheumdis-2016-209730 27965257

[18] Braun J Baraliakos X Deodhar A Poddubnyy D Emery P Delicha EM Secukinumab shows sustained efficacy and low structural progression in ankylosing spondylitis: 4-year results from the MEASURE 1 study. Rheumatol (Oxford) (2019) 58(5):859–68. 10.1093/rheumatology/key375 PMC6477523 30590813

[19] Baraliakos X Kivitz AJ Deodhar AA Braun J Wei JC Delicha EM Long-term effects of interleukin-17A inhibition with secukinumab in active ankylosing spondylitis: 3-year efficacy and safety results from an extension of the Phase 3 MEASURE 1 trial. Clin Exp Rheumatol (2018) 36(1):50–5. 28516874

[20] Marzo-Ortega H Sieper J Kivitz A Blanco R Cohen M Delicha EM Secukinumab provides sustained improvements in the signs and symptoms of active ankylosing spondylitis with high retention rate: 3-year results from the phase III trial, MEASURE 2. RMD Open (2017) 3(2):e000592. 10.1136/rmdopen-2017-000592 29435364 PMC5761290

[21] Pavelka K Kivitz A Dokoupilova E Blanco R Maradiaga M Tahir H Efficacy, safety, and tolerability of secukinumab in patients with active ankylosing spondylitis: a randomized, double-blind phase 3 study, MEASURE 3. Arthritis Res Ther (2017) 19(1):285. 10.1186/s13075-017-1490-y 29273067 PMC5741872

[22] Baraliakos X BJ Deodhar A Poddubnyy D Kivitz AJ Tahir H van Den Bosch F Long-Term Evaluation of Secukinumab in Ankylosing Spondylitis: 5 Year Efficacy and Safety Results from a Phase 3 Trial [abstract]. Arthritis Rheumatol (2018) 70(suppl 10).

[23] Deodhar A Mease PJ McInnes IB Baraliakos X Reich K Blauvelt A Long-term safety of secukinumab in patients with moderate-to-severe plaque psoriasis, psoriatic arthritis, and ankylosing spondylitis: integrated pooled clinical trial and post-marketing surveillance data. Arthritis Res Ther (2019) 21(1):111. 10.1186/s13075-019-1882-2 31046809 PMC6498580

