# Supplementary material for: Secukinumab Demonstrates Sustained Efficacy and Safety in a Taiwanese Subpopulation With Active Ankylosing Spondylitis: Four-Year Results From a Phase 3 Study, MEASURE 1
Source: Front Immunol. 2020 Nov 26;11:561748. doi: 10.3389/fimmu.2020.561748 (PMC7725874; doi:10.3389/fimmu.2020.561748)
Supplement: Supplementary file 1 [file Table_1.docx]

**SUPPLEMENTARY MATERIAL**

**Secukinumab Demonstrates Sustained Efficacy and Safety in a Taiwanese Subpopulation with Active Ankylosing Spondylitis: 4-year Results from a Phase 3 Study, MEASURE 1**

**Jui-Cheng Tseng^1^, James Cheng-Chung Wei^2*^, Atul Deodhar^3^, Ruvie Martin^4^, Brian Porter^4^, Suzanne McCreddin^5^, Zsolt Talloczy^4^**

^1^Kaohsiung Veterans General Hospital, Kaohsiung, Taiwan

^2^Institute of Medicine, Chung Shan Medical University; Department of Medicine, Chung Shan Medical University Hospital; Graduate Institute of Integrated Medicine, China Medical University, Taichung, Taiwan

^3^Oregon Health & Science University, Portland, United States

^4^Novartis Pharmaceuticals Corporation, East Hanover, United States

^5^Novartis Ireland Limited, Dublin, Ireland

**Corresponding author: Dr. James CC Wei**

Institute of Medicine

Chung Shan Medical University

Department of Medicine, Chung Shan Medical University Hospital

Graduate Institute of Integrated Medicine

China Medical University, Taichung, Taiwan

No. 110, Sec. 1, Jianguo N. Rd.,

South District, Taichung, 40201, Taiwan

Contact: +886 4 24739595 #34718

E-mail: [jccwei@gmail.com](mailto:jccwei@gmail.com)

**Table S1. Baseline demographic and clinical characteristics of patients in the overall population and Taiwanese subpopulation**

| **Characteristic** | **Secukinumab 150 mg** | | **Secukinumab 75 mg** | |
| --- | --- | --- | --- | --- |
|  | **Overall population (N = 87)** | **Taiwanese subpopulation (N= 13 )** | **Overall population (N = 100)** | **Taiwanese subpopulation (N= 19)** |
| **Age (years), mean (SD)** | 38.2 (11.6) | 32.6 (9.55) | 42.5 (13.4) | 33.6 (8.53) |
| **Male, n (%)** | 58 (66.7) | 10 (76.9) | 75 (75.0) | 8 (100.0) |
| **Weight (kg), mean (SD)** | 72.5 (15.3) | 67.35 (11.77) | 77.6 (19.3) | 70.50 (13.69) |
| **Total back pain (0–100 mm), mean (SD)** | 62.8 (17.0) | 65.46 (14.89) | 61.1 (19.0) | 56.13 (12.61) |
| **BASDAI (total), mean (SD)** | 6.10 (1.54) | 6.67 (1.17) | 6.04 (1.48) | 5.95 (1.39) |
| **hsCRP (mg/L), median (Min – Max)** | 8.20 (0.2–147.7) | 5.20 (2.2-52.6) | 9.40 (0.4–139.7) | 3.80 (0.2-15.9) |
| **HLA-B27 positive, n (%)** | 61 (70.1) | 13 (100.0) | 81 (81.0) | 8 (100.0) |
| **Mean time since AS diagnosis (years), mean (SD)** | 5.64 (6.42) | 8.38 (4.49) | 7.01 (7.71) | 12.55 (8.61) |
| **Anti–TNF-naïve, n (%)** | 70 (80.5) | 12 ( 92.3) | 76 (76.0) | 7 ( 87.5) |

BASDAI, Bath Ankylosing Spondylitis Disease Activity Index; hsCRP, high sensitivity C-reactive Protein; HLA, Human Leukocyte Antigen; SD, Standard Deviation; TNF, Tumor Necrosis Factor

**Table S2: Comparison of efficacy outcomes of patients originally randomized to secukinumab 150 mg in the overall population *versus* the Taiwanese subpopulation at Week 208**

| **Characteristic** | **Secukinumab 150 mg** | |
| --- | --- | --- |
|  | **Overall Population (N = 87)** | **Taiwanese Subpopulation (N= 13)** |
| **ASAS40 response, n (%)** | 48 (60.8) | 12 (83.3) |
| **ASAS5/6 response, n (%)** | 50 (62.5) | 12 (66.7) |
| **ASAS partial remission, n (%)** | 24 (30.4) | 12 (25) |
| **hsCRP, mean change from baseline (mg/L), mean** ± **SD (n)** | –8.4 ± 26.30 (78) | -10.2 ± 16.92 (12) |
| **BASDAI, mean change from baseline, mean** ± **SD (n)** | –3.4 ± 2.28 (80) | -4.1 ± 1.78 (13) |
| **ASDAS inactive disease, n (%)** | 21 (26.9) | 12 (41.7) |
| **BASFI, mean change from baseline, mean** ± **SD (n)** | –2.9 ± 2.39 (80) | -2.8 ± 2.13 (12) |
| **BASMI, mean change from baseline, mean** ± **SD (n)** | –0.5 ± 1.12 (76) | -0.17 ± 0.56 (12) |
| **SF-36 PCS, mean change from baseline, mean** ± **SD (n)** | 8.2 ± 7.93 (80) | 7.9 ± 6.57 (12) |

Data are presented as observed

N, number of patients randomized; n, number of evaluable patients

hsCRP, high sensitivity C-reactive Protein; ASDAS, Ankylosing Spondylitis Disease Activity Score; SF-36 PCS, Short Form Survey-36 Physical Component Summary; BASFI, Bath Ankylosing Spondylitis Functional Index; BASMI, Bath Ankylosing Spondylitis Metrology Index; BASDAI, Bath Ankylosing Spondylitis Disease Activity Index, ASAS, Assessment of Spondyloarthritis International Society

**Table S3. Clinical improvements with secukinumab through Week 208 (observed data for all patients who entered the extension trial)**

| **Variable** | **Week** | **^*^Any Secukinumab**  **150 mg (N = 21)** | **^^*^Any Secukinumab**  **75 mg (N = 27)** | **Dose-escalated** **to secukinumab 150 mg**  **(N = 13)** |
| --- | --- | --- | --- | --- |
| **ASAS20 response, % (n)** | **52** | 73.7 (19) | 76.0 (25) | NA |
|  | **104** | 85.7 (21) | 76.0 (25) | NA |
|  | **156** | 85.0 (20) | 70.4 (27) | NA |
|  | **208** | 82.4 (17) | 80.8 (26) | 84.6 (13) |
| **ASAS40 response, % (n)** | **52** | 63.2 (19) | 60.0 (25) | NA |
|  | **104** | 61.9 (21) | 44.0 (25) | NA |
|  | **156** | 65.0 (20) | 55.6 (27) | NA |
|  | **208** | 76.5 (17) | 61.5 (26) | 76.9 (13) |
| **ASAS5/6 response, % (n)** | **52** | 63.2 (19) | 52.0 (25) | NA |
|  | **104** | 66.7 (21) | 60.0 (25) | NA |
|  | **156** | 65.0 (20) | 55.6 (27) | NA |
|  | **208** | 64.7 (17) | 76.9 (26) | 84.6 (13) |
| **ASDAS-CRP inactive disease, % (n)** | **52** | 26.3 (19) | 24 (25) | NA |
|  | **104** | 28.6 (21) | 20 (25) | NA |
|  | **156** | 35 (20) | 29.6 (27) | NA |
|  | **208** | 41.2 (17) | 26.9 (26) | 23.1 (13) |
| **BASDAI50 response, % (n)** | **52** | 52.6 (19) | 44.0 (25) | NA |
|  | **104** | 52.4 (21) | 36.0 (25) | NA |
|  | **156** | 50.0 (20) | 40.7 (27) | NA |
|  | **208** | 64.7 (17) | 46.2 (26) | 69.2 (13) |
| **ASAS partial remission, % (n)** | **52** | 21.1 (19) | 20.0 (25) | NA |
|  | **104** | 23.8 (21) | 24.0 (25) | NA |
|  | **156** | 20.0 (20) | 14.8 (27) | NA |
|  | **208** | 23.5 (17) | 19.2 (26) | 23.1 (13) |
| **ASQoL, mean change from baseline ± SD (n)** | **52** | -3.9 ± 5.32 (21) | -3.5 ± 3.30 (27) | NA |
|  | **104** | -4.4 ± 5.33 (21) | -4.1 ± 3.74 (25) | NA |
|  | **156** | NA | NA | NA |
|  | **208** | NA | NA | NA |
| **hsCRP, mean change from baseline ± SD (n)** | **52** | -7.67 ± 13.69 (19) | -7.08 ± 10.98 (25) | NA |
|  | **104** | -7.49 ± 12.11 (21) | -6.55 ± 10.31 (25) | NA |
|  | **156** | -8.09 ± 11.78 (20) | -6.52 ± 11.22 (27) | NA |
|  | **208** | -9.34 ± 15.04 (17) | -8.05 ± 13.57 (26) | -10.68 ± 13.6 (13) |
| **BASDAI, mean change from baseline ± SD (n)** | **52** | -3.23 ± 1.81 (19) | -2.68 ± 1.85 (25) | NA |
|  | **104** | -3.37 ± 1.88 (21) | -2.62 ± 1.66 (25) | NA |
|  | **156** | -3.58 ± 1.67 (20) | -2.68 ± 1.76 (27) | NA |
|  | **208** | -3.76 ± 2.01 (17) | -2.93 ± 1.54 (26) | -3.6 ± 1.36 (13) |
| **SF-36 PCS,** **mean change from baseline ± SD (n)** | **52** | 7.50 ± 6.72 (21) | 6.89 ± 4.71 (27) | NA |
|  | **104** | 7.82 ± 8.15 (21) | 7.66 ± 4.78 (25) | NA |
|  | **156** | 7.42 ± 7.66 (20) | 7.16 ± 4.97 (27) | NA |
|  | **208** | 7.40 ± 7.31 (17) | 8.36 ± 5.14 (26) | 9.08 ± 4.91 (13) |
| **MASES, mean change from baseline± SD (n)** | **52** | -1.0 **±** 1.22 (21) | -0.7 **±** 1.98 (27) | NA |
|  | **104** | -1.1 **±** 1.61 (21) | -0.6 **±** 2.63 (25) | NA |
|  | **156** | -0.4 **±** 1.27 (20) | -1.0 **±** 1.81 (27) | NA |
|  | **208** | -0.7 **±** 1.10 (17) | -1.1 **±** 2.17 (26) | -1.1 **±** 1.89 (13) |
| **BASFI, mean change from baseline± SD (n)** | **52** | -2.2 **±** 2.04 (19) | -2.2 **±** 1.76 (25) | NA |
|  | **104** | -2.7 **±** 1.86 (21) | -2.1 **±** 1.88 (25) | NA |
|  | **156** | -2.6 **±** 1.94 (20) | -2.2 **±** 1.87 (27) | NA |
|  | **208** | -2.5 **±** 1.99 (17) | -2.2 **±** 1.75 (26) | -2.5 **±** 2.18 (13) |
| **BASMI, mean change from baseline± SD (n)** | **52** | -0.4 **±** 0.57 (19) | -0.3 **±** 0.83 (25) | NA |
|  | **104** | -0.2 **±** 0.67 (21) | -0.3 **±** 0.81 (25) | NA |
|  | **156** | -0.3 **±** 0.61 (20) | -0.2 **±** 0.82 (27) | NA |
|  | **208** | -0.2 **±** 0.47 (17) | -0.2 **±** 0.95 (26) | -0.4 **±** 0.96 (13) |
| **FACIT-Fatigue, mean change from baseline± SD (n)** | **52** | 8.2 **±** 11.08 (21) | 7.7 ± 6.59 (27) | NA |
|  | **104** | 10.8 **±** 9.69 (21) | 8.6 **±** 7.35 (25) | NA |
|  | **156** | 10.2 **±** 10.9 (20) | 8.0 **±** 8.68 (27) | NA |
|  | **208** | 10.4 **±** 11.05 (17) | 9.6 **±** 7.44 (26) | 10.8 **±** 6.66 (13) |

N, number of patients in the extension trial; n, number of evaluable patients;

SD, standard deviation; NA, not assessed (data not collected)/not applicable

^*^Secukinumab 150 and 75 mg groups included placebo patients re-randomized to secukinumab 150 or 75 mg at Week 16 or 24.

^^^At Week 208, Secukinumab 75 mg group included patients (N = 13) who were dose-escalated from 75 mg to 150 mg

hsCRP, high sensitivity C-reactive Protein; ASDAS, Ankylosing Spondylitis Disease Activity Score; SF-36 PCS, Short Form Survey-36 Physical Component Summary; ASQoL, Ankylosing Spondylitis Quality of Life; MASES, Maastricht Ankylosing Spondylitis Enthesitis Score; BASFI, Bath Ankylosing Spondylitis Functional Index; BASMI, Bath Ankylosing Spondylitis Metrology Index; FACIT, Functional Assessment of Chronic Illness Therapy; BASDAI, Bath Ankylosing Spondylitis Disease Activity Index, ASAS, Assessment of Spondyloarthritis International Society
